# Just Plain Hot: Examining Summer Daily High Heat Indices and Community-Level Social Vulnerability on Emergency Medical Services On-Scene Responses in San Antonio, Texas, 2015-2018

**DOI:** 10.7759/cureus.39914

**Published:** 2023-06-03

**Authors:** Lisa K Zottarelli, Xiaohe Xu, Thankam S Sunil, Shamatanni Chowdhury

**Affiliations:** 1 Social Work, University of Tennessee, Knoxville, Knoxville, USA; 2 Sociology, University of Texas at San Antonio, San Antonio, USA; 3 Public Health, University of Tennessee, Knoxville, Knoxville, USA

**Keywords:** morbidity, ems, geographic location, heat, social vulnerability

## Abstract

Introduction

Increased summer heat has a deleterious effect on people’s health and the healthcare system. Emergency Medical Services (EMS) are at the healthcare system frontline, responsive to the community and environmental conditions. The present study examined how EMS on-scene response is affected by community-level social vulnerability and heat.

Methods

The Centers for Disease Control and Prevention’s Social Vulnerability Index, heat and humidity data from the National Weather Service, and City of San Antonio EMS data were collected. Data were analyzed using negative binomial regression models with time-stratified case-crossover design to observe independent and interactive effects of heat and social vulnerability on EMS on-scene response over four constricted calendar years.

Results

Results indicated that community-based social vulnerability and heat, independently and interactively, are associated with increased EMS on-scene responses.

Conclusion

Even when examining normal summertime heat conditions, there is evidence of the relationships between geographic and environmental conditions and the healthcare system.

## Introduction

Extreme heat is associated with increased mortality and morbidity, especially for vulnerable populations such as people who are elderly, have lower income, and are socially isolated [1-3]. It can strain and even disrupt health-sustaining infrastructure such as electrical grids and healthcare systems [4]. While extreme heat gets attention, even seemingly normal summertime high temperatures can negatively affect people’s health and the healthcare infrastructure [5,6]. While extreme heat has gained attention as a hazard, the trend of overall increases in normal summertime heat needs more attention as it presents a potentially less recognized threat. The aim of this study was to examine how community morbidity is affected by normal summer hot weather with the context of community-level social vulnerability in a city where hot summers are expected. It adds to the literature on the impact of warming temperatures on vulnerable population in urban settings and provides insight into the potential for healthcare infrastructure stress due to summer heat.

Emergency Medical Services (EMS) provide a means to examine community morbidity at the frontline of the healthcare infrastructure [7,8]. EMS are “uniquely mobile” [7] and respond to people’s medical needs in the community. As with many social phenomena, EMS calls are not evenly nor randomly distributed across a city or over time. Ambulance use has been associated with variations in patient health and socioeconomic status [9]. Higher proportions of Medicaid and uninsured patients [10,11], as well as elderly and critically ill patients [11], arrive at the emergency department by ambulance. Additionally, the location of EMS on-scene response and transport distance to a medical facility impacts patient outcomes. For example, a cross-sectional study of 16,082 adults found an 8% increase in the odds of death for every 5-mile increase in distance to the nearest trauma center [12]. Further, there was a 3% increase in the odds of death for every five-year increase in neighborhood median age, while the odds of death decrease by 27% when neighborhood annual per capita income was greater than $25,000 [12].

Heat has been found to increase the utilization of pre-hospital EMS [5,6,13-16]. In Boston, a four-year study found that a 90°F or higher maximum daily temperature was associated with an increase in police, fire, and EMS dispatch calls [6]. In Western Pennsylvania, the number of EMS on-scene responses increased as the temperature increased [5]. In a summer 2005 study conducted in Toronto, there were increased heat-related illness ambulance calls associated with increased temperature means and increased temperature maximums [13]. In Brisbane, extreme high temperatures, measured hourly, were associated with increased ambulance call volume, and the increased call volume lasted 24 hours after the extreme high temperature [15]. Extreme heat and heat waves were associated with an increased number of ambulance dispatches in Huanina [14]. These studies point to a relationship between hotter-than-normal temperatures and EMS utilization, but they have been conducted in urban areas that tend to experience moderate summer heat and often lack significant private air conditioning infrastructure.

Heat risk is based on the temperatures above the normal in a location, often considering time of year [17], and normal temperatures change over time. The official normal or average temperatures are adjusted every decade to reflect the previous three decades of data. The current temperature references are calibrated to 1991-2020 and show a general upward shift in temperature compared to previous annual averages [18]. As normal temperatures shift upward, “above average” temperatures become fewer even though the actual temperatures are higher [19]. Therefore, it can be expected that there will be higher EMS utilization on hot days, even when those daily high temperatures are within the normal temperature range.

The risks associated with heat have both individual and spatial dimensions. Health conditions, behaviors, and living conditions may place individuals at risk to heat as a hazard at a lower temperature threshold compared to the general populations or other people within the same household. Some of the individual risk can be exacerbated given the spatial dimensions of risk. For example, people may decide not to use air conditioning as a heat mitigation tool because of the cost or may lack private transportation and have greater exposure to heat while walking and waiting for the bus. In urban areas, such as San Antonio, people who may have greater individual risk often live near others with similar risks due to the patterns of residential segregation. This results in areas of a city where there are high concentrations of vulnerable populations [20].

The influence of extreme heat events on EMS is embedded within a context-specific environment characterized by adaptive capacity and vulnerability [21]. There is differential exposure to deleterious conditions in areas of the city that have unmet needs and/or greater needs for services [22]. A city is not uniform in its population nor services, rather the built environment has spatial dimensions that are complex and dynamic results of historic and contemporary inequality. Community inequalities expose vulnerability to multiple hazards, and community-level social vulnerability impacts delivery of care and care outcomes. As EMS is a resource allocated by the city or county government to provide services to people in need, EMS is responsive to the built environment and social conditions.

Spatial differences to heat vulnerability have been identified, often with inner cities having the greatest heat vulnerability [3]. Ambulance call volume increased by 10% during extreme heat days compared to normal temperature days and spatial “hot spots” for EMS calls were identified in industrial and recreational areas [22]. During extreme heat events in Phoenix, areas with a higher proportion of residents living alone, renting, Black, Hispanic, and linguistically isolated, had higher numbers of heat distress calls to emergency services dispatch [23]. The incidences of heat-related illness were greater on hot days in areas of downtown, especially lower-income areas and neighborhoods with higher rates of homelessness, as well as in recreational areas [24].

The public experiences extreme heat as a local phenomenon and it is responded to within a local context. EMS in the U.S. are operated by local government. Further, EMS can be structured in different ways including subcontracting to third-party or privatized ambulance services, as a unit within a local Fire Department, or as a stand-alone division within the city or county government [25]. Social vulnerability also has a local and spatial dimension where areas of a city or county have greater proportions of the population with characteristics that place them at heat health risk (e.g., higher proportions of elderly, greater concentrations of lower-income households, etc.) [26]. Given that heat, EMS structure, and social vulnerability are experienced at a local level, the relationship of heat, community-level social vulnerability, and EMS utilization in the U.S. requires analysis at the city or county level. 

In San Antonio in 2018, EMS incidents increased in areas of the city with greater overall social vulnerability and on higher temperature days [27]. Yet, it is not clear if this year was an exception or part of a larger pattern of summertime EMS utilization. Other locations where health and heat events have been studied over multiple years include King County, Washington [28], Milwaukee, Wisconsin [29], and Phoenix, Arizona [30]. This study adds to the literature by examining the relationship between community-level social vulnerability and heat indices on EMS on-scene response over multiple years. Multi-year studies are important because they allow for the inclusion of year-to-year variations and to ensure that other factors beyond weather do not unduly influence the results.

San Antonio, Texas

San Antonio, Texas, is in south central Texas and is the seventh most populous city in the U.S. It has a humid subtropical climate; summers are expected to be hot and daily summertime high temperatures above 90ºF are common. San Antonio has significant heat-mitigating infrastructure including a high prevalence of air conditioning availability in private homes, schools, libraries, and businesses. San Antonio also has some of the highest levels of income segregation by residential location in the U.S., with significant impacts on access to services and socioeconomic outcomes [31]. It is estimated that 17.8% of people live in poverty and 18.7% of people under 65 years of age lack health insurance [32]. 

At the time of this study, Emergency Medical Services within the city were provided through the San Antonio Fire Department under the command of an Assistant Fire Chief. In addition to the Assistant Fire Chief, there were 21 supervisory staff and 371 Emergency Medical Technician-Paramedics with 33 full-time ambulances and eight peak-period ambulances [33]. The ambulance units respond to Basic Life Support (BLS) and Advanced Life Support (ALS) calls and are positioned at fire stations throughout the city.

In addition to location, time is an important contextual element in studying heat, social vulnerability, and EMS. Many studies limit or constrain the months included in analysis to focus on the hottest months of the year [28]. A constrained summer, from May 1 to September 30, is used in this study. In San Antonio, it is often hot enough to have extreme heat days starting in April but there is a multi-week city-wide celebration, Fiesta, that includes parades, outdoor festivals, and other events. The length of Fiesta varies depending on when Easter falls during that month. EMS is very active during Fiesta and the dispersion of events throughout the city affects the spatial distribution of calls. Therefore, April was excluded from the analysis. 

## Materials and methods

The present study is a quantitative time-stratified case-crossover examination of heat and community-level social vulnerability on EMS on-scene responses. Data were analyzed for four constrained calendar years from May 1 to September 30, 2015 to 2018. Sixty-one zip codes were included in the analysis of 2015 and 63 zip codes were included in the analysis of 2016 to 2018. The five-month constrained calendar has 162 days per year for a total of 648 days over the four-year period. Three hypotheses were tested in this study: H1: Zip code areas with greater community-level social vulnerability will have a higher number of EMS on-scene responses, H2: Higher heat days will have a greater number of EMS on-scene responses, and H3: The interaction between community-level social vulnerability and the heat index will increase the number of EMS on-scene responses. 

Variables 

EMS On-Scene Response

The EMS on-scene response refers to an event where EMS was dispatched to an incident and the EMS personnel made contact with a patient regardless of how the call was received by dispatch or if the patient was transported to the hospital or not. The EMS on-scene response data are from the City of San Antonio and were provided by zip code by day. In total, 63 zip codes were included in the dataset. 

Heat

Heat can be measured in several ways. For this study, we used the Heat Index (HI) as the measure of heat. The HI is calculated using temperature and humidity data. This approach to measuring heat accounts for the impact of temperature coupled with humidity on human physical functioning, especially maintaining body temperature through perspiration and evaporation. Additionally, the HI is routinely communicated to the public during the local news shows and is used in the city of San Antonio’s Heat Plan [34]. The data to calculate the HI were from the National Oceanic and Atmospheric Administration National Oceanic and Atmospheric Administration National Centers for Environmental Information [35] and the HI calculation is from the National Weather Service Weather Prediction Center [36]. The highest HI per date were used in this study. 

Community-Level Social Vulnerability

The community-level social vulnerability data were from the Centers for Disease Control and Prevention/Agency for Toxic Substances and Disease Registry (CDC/ATSDR) Social Vulnerability Index (SVI) [37]. The data were converted from census tract to zip code to align with the EMS data. Further discussion of the data and operationalization of variables are provided in Zottarelli et al. (2021) [27].

## Results

**Figure 1:**
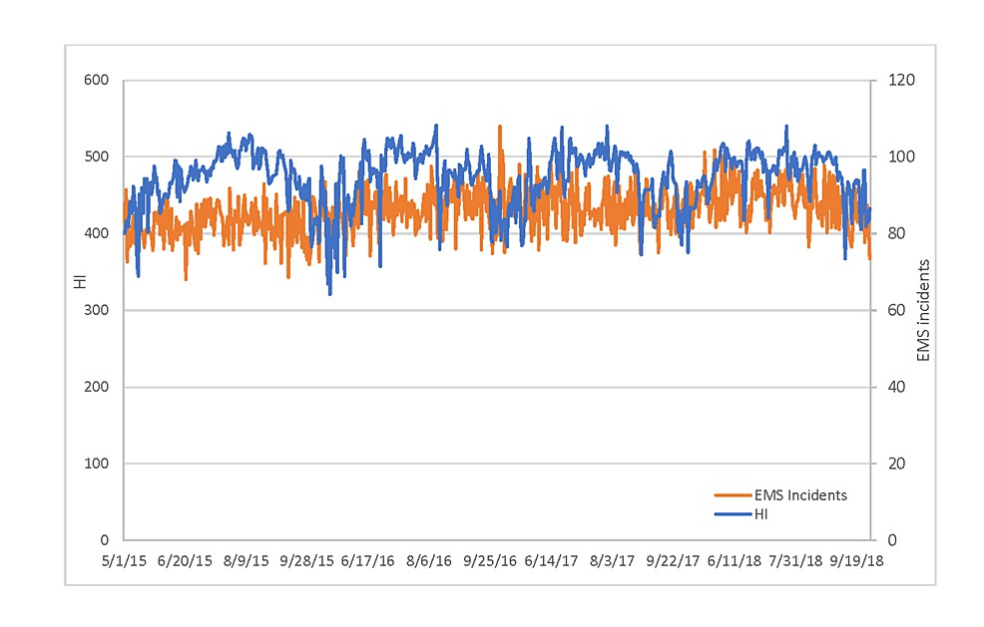
Trends in Heat Index and EMS Calls Over Four Constricted Summers, 2015-2018 EMS: Emergency Medical Services, HI: heat index

Figure 1 captures the total EMS call volume across all zip codes for each day and the daily heat index for the study period, May 1 to September 30, 2015-2018. The daily HI high ranged from a low daily high HI of 64.11^o^F in May 2016 to a high daily high HI of 108.28^o^F in August 2016. The lowest daily mean HI high was 83.95^o^F in May 2016 and the highest daily mean HI high was 101.15^o^F in August 2015. The highest yearly mean HI was 95.59^o^F in 2018. Over the 612 days in the study, total EMS call volume ranged from a low of 340 calls to a high of 540 with a mean of 430.21 (sd = 29.09). In general, over the four constricted summers, EMS call volumes are higher during high HI days. 

At the zip code level, as shown in Table 1, the number of EMS on-scene responses by zip code by date ranged from 0 to 65 calls. The lowest monthly mean of EMS on-scene response by zip code by date was 6.53 calls (sd = 6.50) in May 2015. The highest monthly mean of 7.41 on-scene responses (sd = 7.33) was in July 2018. The yearly number of EMS on-scene responses by zip code by date varied from a mean of 6.62 (sd = 6.60) in 2015 to 7.25 (sd = 6.99) in 2018. The SVI ranged from 0.06 to 0.97 with a mean of 0.55 and a standard deviation of 0.27. 

**Table 1:** Sample Characteristics by Year EMS: Emergency Medical Services, SVI: Social Vulnerability Index

		N	Minimum	Maximum	Mean	SD
2015						
May	# of EMS Incidents	1,922	0	47	6.53	6.50
	Heat Index	1,922	68.91	97.51	87.42	6.72
	SVI	1,922	0.06	0.97	0.55	0.27
June	# of EMS Incidents	1,860	0	48	6.56	6.46
	Heat Index	1,860	86.46	99.10	93.25	3.42
	SVI	1,860	0.06	0.97	0.55	0.27
July	# of EMS Incidents	1,922	0	44	6.76	6.85
	Heat Index	1,922	93.60	106.28	99.06	3.09
	SVI	1,922	0.06	0.97	0.55	0.27
August	# of EMS Incidents	1,922	0	44	6.72	6.71
	Heat Index	1,922	93.00	105.86	100.15	3.69
	SVI	1,922	0.06	0.97	0.55	0.27
September	# of EMS Incidents	1,860	0	43	6.51	6.45
	Heat Index	1,860	85.84	101.71	94.39	4.20
	SVI	1,860	0.06	0.97	0.55	0.27
2015 Combined	# of EMS Incidents	9,486	0	48	6.62	6.60
	Heat Index	9,486	68.91	106.28	94.87	6.37
	SVI	9,486	0.06	0.97	0.55	0.27
2016						
May	# of EMS Incidents	1,891	0	45	6.84	6.59
	Heat Index	1,891	64.11	100.31	83.95	9.57
	SVI	1,891	0.06	0.97	0.54	0.27
June	# of EMS Incidents	1,830	0	52	7.07	6.87
	Heat Index	1,830	71.34	104.51	93.63	7.13
	SVI	1,830	0.06	0.97	0.54	0.27
July	# of EMS Incidents	1,891	0	46	7.08	7.04
	Heat Index	1,891	94.33	105.60	101.00	2.61
	SVI	1,891	0.06	0.97	0.54	0.27
August	# of EMS Incidents	1,891	0	49	7.15	6.99
	Heat Index	1,891	75.93	108.28	96.63	6.41
	SVI	1,891	0.06	0.97	0.54	0.27
September	# of EMS Incidents	1,830	0	46	7.15	7.08
	Heat Index	1,830	77.79	102.73	94.23	6.17
	SVI	1,830	0.06	0.97	0.54	0.27
2016 Combined	# of EMS Incidents	9,333	0	52	7.06	6.92
	Heat Index	9,333	64.11	108.28	93.89	8.80
	SVI	9,333	0.06	0.97	0.54	0.27
2017						
May	# of EMS Incidents	1,891	0	50	7.25	6.88
	Heat Index	1,891	76.56	104.86	86.84	6.64
	SVI	1,891	0.06	0.97	0.55	0.27
June	# of EMS Incidents	1,830	0	46	7.25	6.97
	Heat Index	1,830	89.42	107.70	96.19	5.01
	SVI	1,830	0.06	0.97	0.55	0.27
July	# of EMS Incidents	1,891	0	47	7.07	6.82
	Heat Index	1,891	95.09	108.09	100.34	2.34
	SVI	1,891	0.06	0.97	0.55	0.27
August	# of EMS Incidents	1,891	0	52	7.04	6.87
	Heat Index	1,891	74.42	102.86	96.11	6.39
	SVI	1,891	0.06	0.97	0.55	0.27
September	# of EMS Incidents	1,830	0	40	7.04	6.73
	Heat Index	1,830	77.08	101.48	89.88	6.10
	SVI	1,830	0.06	0.97	0.55	0.27
2017 Combined	# of EMS Incidents	9,333	0	52	7.13	6.85
	Heat Index	9,333	74.42	108.09	93.88	7.36
	SVI	9,333	0.06	0.97	0.55	0.27
2018						
May	# of EMS Incidents	1,891	0	43	7.27	6.83
	Heat Index	1,891	75.03	102.33	91.61	5.68
	SVI	1,891	0.06	0.97	0.55	0.27
June	# of EMS Incidents	1,830	0	51	7.36	7.09
	Heat Index	1,830	83.45	104.17	99.17	4.13
	SVI	1,830	0.06	0.97	0.55	0.27
July	# of EMS Incidents	1,891	0	65	7.41	7.33
	Heat Index	1,891	84.10	108.04	98.36	3.97
	SVI	1,891	0.06	0.97	0.55	0.27
August	# of EMS Incidents	1,891	0	50	7.29	7.06
	Heat Index	1,891	88.39	102.95	98.40	2.97
	SVI	1,891	0.06	0.97	0.55	0.27
September	# of EMS Incidents	1,830	0	46	6.92	6.62
	Heat Index	1,830	73.38	99.90	90.34	6.13
	SVI	1,830	0.06	0.97	0.55	0.27
2018 Combined	# of EMS Incidents	9,333	0	65	7.25	6.99
	Heat Index	9,333	73.38	108.04	95.59	6.04
	SVI	9,333	0.06	0.97	0.55	0.27

To study the effects of factors in predicting the number of EMS on-scene responses, three time-stratified case-crossover negative binomial regression models [38,39] were estimated to examine the independent effects of HI and community-level SVI, as well as the interaction effect of HI and SVI on EMS on-scene response. Given the count nature of the dependent variable, number of EMS on-scene responses, with overdispersion, we conducted negative binomial regression analyses. While not reported in the table, we used three-way interactions between year, month, and day of the week. The negative binomial regression models with the time-stratified case-crossover design are equivalent to time-series methods and can account for residual autocorrelation and overdispersion [35,36]. Since the time-series data used in this study are zip code level data, intra-zip code area correlation was allowed by specifying the clustered SEs. In Table 2, Model 1 shows the effects of HI, the effects of SVI after controlling for HI are found in Model 2, and Model 3 presents the multiplicative of HI and SVI. All three models were controlled for time strata dummy variables, i.e., three-way interactions among year, month, and day of the week. We also reported incidence rate ratio and 95% CI for all the coefficients.

**Table 2:** Time-Stratified Case-Crossover Negative Binomial Regression Models to Predict EMS Calls, 2015-2018 EMS: Emergency Medical Services, SVI: Social Vulnerability Index, HI: heat index, IRR: incidence rate ratio

Variable	Model 1 (n=37,485)		Model 2 (n=37,485)		Model 3 (n=37,485)	
	IRR	95% CI	IRR	95% CI	IRR	95% CI
Constant	5.672***	4.582, 7.022	2.331***	1.416, 3.838	2.346***	1.425, 3.860
Heat Index	1.002**	1.001, 1.003	1.001***	1.000, 1.002	1.001**	1.000, 1.002
SV Index			4.61***	2.066, 10.288	4.61***	2.066, 10.287
HI*SVI					1.004*	1.000, 1.008
*p<0.05; **p<0.01; **p<0.001						

Model 1 shows that for every unit increase in HI, the number of EMS on-scene response calls is expected to increase by a factor of 1.002 and is statistically significant (p<0.01). When controlled for HI and the time stratification variables, SVI is statistically and positively associated with EMS on-scene responses. That is, for every unit increase in SVI, the rate of EMS on-scene responses is expected to increase by a factor of 4.61 (p<0.001). While Model 3 showed similar effect for SVI in predicting EMS on-scene responses, there is a significant interaction between HI and SVI, indicating that zip codes where social vulnerability is more prevalent tend to have even higher EMS on-scene responses holding the time stratification variables constant. 

## Discussion

The principal finding of this study is that community-level social vulnerability was associated with an increase in the number of Emergency Medical Service on-scene responses. Social vulnerability captures inequality in a complex and multifaceted way and can identify differential exposure to hazardous conditions [40]. Community inequality is intersectional and geographically clustered, with residents often lacking access to resources and services. Further, urban areas are more vulnerable to the negative health effects of extreme heat due to infrastructure dependency, built environment, and greater density of people [21]. For the public, geographic differences in ambulance utilization have been shown to be associated with patient health conditions and outcomes, separate from the actual medical condition of the patient [9,12,22,23]. 

Spatial inequality within a city creates vulnerability that increases the need for EMS response. The CDC/ATSDR Social Vulnerability Index was developed to identify areas that may need greater disaster support [37]. It is constructed using multiple dimensions of inequality that includes factors such as unemployment, minority status, disability, language, housing, etc. It is designed to be used by city and county officials and others responsible for disaster and emergency preparedness, response, and recovery. Based on this study, the SVI can be used to inform disaster preparedness. Baseline EMS on-screen response over the four summers was higher in areas of the city with greater social vulnerability. Higher heat indexes interacted with social vulnerability to increase the number of EMS on-scene responses. The baseline stressing of the EMS system in what is becoming more routine heat points to preparedness needs for heat and extreme heat events. 

The relationship between the heat index and EMS on-scene response is complicated. The importance of the heat index on increasing the number of EMS on-scene responses, while statistically significant, had a small effect alone. This raises more questions than it answers. One important consideration would be to continue to examine heat and extreme heat, in the various ways it is measured (e.g., daytime temperature, nighttime temperatures, heat waves, extreme heat etc.), to determine how to better capture the relationship between heat and EMS. In a city where people expect hot summers and where heat-mitigating infrastructure is ubiquitous, it may be that heat is a more complex factor than captured in this study. Nevertheless, the results suggest a relationship that warrants further examination in future studies. 

SVI were available by census tract and the EMS data were provided by zip code. The authors converted the SVI data to zip code to align to the EMS data. The Housing and Urban Development (HUD) USPS Zip Code Crosswalk [41] was used for the conversion. While this approach is systematic and standardized, converting the data introduces the possibility of misalignment of the distribution based on an assumed physical dispersion within the original census tract. Despite the issue of dispersion, it was important to convert to zip code, as this is a unit in which the local government captures data and allocates resources within its 10 council districts. 

The EMS data used for this study were raw numbers by zip code by day. No differentiation by call type (i.e., ALS or BLS), disposition of call (e.g., transport to hospital), length of time on scene, time of day of call, or any other call details were provided. The overall number of calls is meaningful for planning about general trends, but more information about types and characteristics of the calls, access to healthcare services and emergency department patient discharge data could provide additional understanding of the impact of environmental and social factors on EMS. 

There are implications for the EMS workforce. In the U.S., EMS are pre-hospital services responding to medical needs in the community that occur in mobile space outside of hospitals, doctor’s offices, and clinics. They respond to calls, often made by the public to the emergency response system. EMS personnel are exposed to weather conditions, including heat, and during many calls may perform medical assessments and interventions on a street, sidewalk, or other outside location. For the EMS workforce, more calls can mean more time on calls with less time to recover from heat exposure and less time to engage in heat illness mitigation efforts such as drinking water to maintain hydration. This has important implications for the public, EMS workers, as well as municipal emergency response practices, especially with increase in normal summer temperatures in cities like San Antonio, Texas. Better understanding of how heat affects calls and how this may, in turn, affect workers, could support safer working conditions and better EMS response.

This study location is one of the most populous cities in the U.S. but has not been examined in terms of heat and social vulnerability. Findings related to relationship between heat and Emergency Medical Services are consistent with previous studies conducted in different climatic zones, such as Toronto [13,22,24], King County Washington [28], and Brisbane [15] among other cities. Future studies should be done to determine the applicability of the findings across cities with different socioeconomic and environmental conditions. It is also important to note that geospatial analysis of small area data, such as our zip code areas in a major metropolitan area in the U.S., can help identify sociodemographic and geographic variations in health disparities. Our analysis of the San Antonio zip code areas can enhance the growing body of research on the spatial distribution of social vulnerability and its association with environmental exposure-related health outcomes. Understanding the relationship between social vulnerability and the heat index on EMS has the potential to improve identification of community need as well as support for first responders during high heat conditions. It also provides baseline information for extreme heat preparedness and response. 

## Conclusions

Cities such as San Antonio, Texas, with historical and contemporary spatially identifiable inequality, will have to plan not only for extreme heat events but also the general upward trend in normal summertime heat and its differential impacts on the local population. EMS are on the healthcare system frontline and are both exposed to heat and sensitive to the impact of heat on the community. Understanding the relationship between community and environmental conditions on a frontline component of the healthcare system is of critical importance as the growing impacts of climate change and inequality become more apparent.

## References

[1] Gronlund CJ, Berrocal VJ, White-Newsome JL, Conlon KC, O'Neill MS (2015). Vulnerability to extreme heat by socio-demographic characteristics and area green space among the elderly in Michigan, 1990-2007. Environ Res.

[2] Klinenberg E (2015). Heat Wave: A Social Autopsy of Disaster in Chicago. https://press.uchicago.edu/ucp/books/book/chicago/H/bo20809880.html.

[3] Reid CE, O'Neill MS, Gronlund CJ, Brines SJ, Brown DG, Diez-Roux AV, Schwartz J (2009). Mapping community determinants of heat vulnerability. Environ Health Perspect.

[4] Jones HM, Mccray EL, Birlek SD (2019). Understanding decision context to improve heat health information. Bull Am Meteorol Soc.

[5] Ramgopal S, Dunnick J, Owusu-Ansah S, Siripong N, Salcido DD, Martin-Gill C (2019). Weather and temporal factors associated with use of emergency medical services. Prehosp Emerg Care.

[6] Williams AA, Allen JG, Catalano PJ, Buonocore JJ, Spengler JD (2020). The influence of heat on daily police, medical, and fire dispatches in Boston, Massachusetts: relative risk and time-series analyses. Am J Public Health.

[7] Bigham BL, Kennedy SM, Drennan I, Morrison LJ (2013). Expanding paramedic scope of practice in the community: a systematic review of the literature. Prehosp Emerg Care.

[8] Weisskopf MG, Anderson HA, Foldy S, Hanrahan LP, Blair K, Török TJ, Rumm PD (2002). Heat wave morbidity and mortality, Milwaukee, Wis, 1999 vs 1995: an improved response?. Am J Public Health.

[9] Hanchate AD, Paasche-Orlow MK, Dyer KS, Baker WE, Feng C, Feldman J (2017). Geographic variation in use of ambulance transport to the emergency department. Ann Emerg Med.

[10] Meisel ZF, Pines JM, Polsky D, Metlay JP, Neuman MD, Branas CC (2011). Variations in ambulance use in the United States: the role of health insurance. Acad Emerg Med.

[11] Squire BT, Tamayo A, Tamayo-Sarver JH (2010). At-risk populations and the critically ill rely disproportionately on ambulance transport to emergency departments. Ann Emerg Med.

[12] Jarman MP, Curriero FC, Haut ER, Pollack Porter K, Castillo RC (2018). Associations of distance to trauma care, community income, and neighborhood median age with rates of injury mortality. JAMA Surg.

[13] Bassil KL, Cole DC, Moineddin R, Craig AM, Lou WY, Schwartz B, Rea E (2009). Temporal and spatial variation of heat-related illness using 911 medical dispatch data. Environ Res.

[14] Cheng J, Xu Z, Zhao D, Xie M, Zhang H, Wang S, Su H (2016). The burden of extreme heat and heatwave on emergency ambulance dispatches: a time-series study in Huainan, China. Sci Total Environ.

[15] Guo Y (2017). Hourly associations between heat and ambulance calls. Environ Pollut.

[16] Kue RC, Dyer KS (2013). The impact of heat waves on transport volumes in an urban emergency medical services system: a retrospective review. Prehosp Disaster Med.

[17] (2023). Heat Forecast Tools. https://www.weather.gov/safety/heat-index.

[18] (2021). NOAA Delivers New U.S. Climate Normals. https://www.ncei.noaa.gov/news/noaa-delivers-new-us-climate-normals.

[19] (2021). NOAA’s Updated U.S. Climate Data Will Establish “New Normal”. https://www.ncei.noaa.gov/news/Upcoming-NOAA-2020-Climate-Normals.

[20] Seong K, Jiao J, Mandalapu A (2022). Evaluating the effects of heat vulnerability on heat-related emergency medical service incidents: lessons from Austin, Texas. Environ Plan B Urban Anal City Sci.

[21] Ellena M, Breil M, Soriani S (2020). The heat-health nexus: systematic literature review exploring the socio-economic vulnerabilities and built environment characteristics. Urban Clim.

[22] Dolney TJ, Sheridan SC (2006). The relationship between extreme heat and ambulance response calls for the city of Toronto, Ontario, Canada. Environ Res.

[23] Uejio CK, Wilhelmi OV, Golden JS, Mills DM, Gulino SP, Samenow JP (2011). Intra-urban societal vulnerability to extreme heat: the role of heat exposure and the built environment, socioeconomics, and neighborhood stability. Health Place.

[24] Bassil KL, Cole DC, Moineddin R, Lou W, Craig AM, Schwartz B, Rea E (2011). The relationship between temperature and ambulance response calls for heat-related illness in Toronto, Ontario, 2005. J Epidemiol Community Health.

[25] Seim J (2017). The ambulance: toward a labor theory of poverty governance. Am Sociol Rev.

[26] Zottarelli LK, Blake SA, Garza MT (2022). Communicating heat-health information to the public: assessing municipal government extreme heat event website content. Weather Clim Soc.

[27] Zottarelli LK, Sharif HO, Xu X, Sunil TS (2021). Effects of social vulnerability and heat index on emergency medical service incidents in San Antonio, Texas, in 2018. J Epidemiol Community Health.

[28] Calkins MM, Isaksen TB, Stubbs BA, Yost MG, Fenske RA (2016). Impacts of extreme heat on emergency medical service calls in King County, Washington, 2007-2012: relative risk and time series analyses of basic and advanced life support. Environ Health.

[29] DeVine AC, Vu PT, Yost MG, Seto EY, Busch Isaksen TM (2017). A geographic analysis of emergency medical service calls and extreme heat in King County, WA, USA (2007-2012). Int J Environ Res Public Health.

[30] Golden JS, Hartz D, Brazel A, Luber G, Phelan P (2008). A biometeorology study of climate and heat-related morbidity in Phoenix from 2001 to 2006. Int J Biometeorol.

[31] Yi H, Kreuter UP, Han D, Güneralp B (2019). Social segregation of ecosystem services delivery in the San Antonio region, Texas, through 2050. Sci Total Environ.

[32] (2022). QuickFacts San Antonio city, Texas. https://www.census.gov/quickfacts/fact/table/sanantoniocitytexas/RHI125219.

[33] City of San Antonio (2020 (2020). San Antonio Fire Department Emergency Medical Services (EMS). https://www.sanantonio.gov/SAFD/About/Divisions/Emerency-Medical-Services.

[34] (2022). City of San Antonio (2020-2022) Heat Plan. https://www.sanantonio.gov/Health/EmergencyManagement/heatplan.

[35] (2020). Data Tools: Local Climatological Data (LCD), 2019. https://www.ncdc.noaa.gov/cdo-web/datatools/lcd.

[36] National Weather Service Weather Prediction Center. (2014 (2014). The Heat Index Equation. https://www.wpc.ncep.noaa.gov/html/heatindex_equation.shtml.

[37] (2021). CDC/ATSDR Social Vulnerability Index. https://www.atsdr.cdc.gov/placeandhealth/svi/index.html.

[38] Lu Y, Zeger SL (2007). On the equivalence of case-crossover and time series methods in environmental epidemiology. Biostatistics.

[39] Armstrong BG, Gasparrini A, Tobias A (2014). Conditional Poisson models: a flexible alternative to conditional logistic case cross-over analysis. BMC Med Res Methodol.

[40] Galea S, Freudenberg N, Vlahov D (2005). Cities and population health. Soc Sci Med.

[41] (2019). HUD USPS ZIP Code Crosswalk Files. HUD-USPS Crosswalk Files.

